# Supplementary material for: Beyond surface tension-dominated water surface jumping
Source: Nat Commun. 2025 Mar 28;16:3034. doi: 10.1038/s41467-025-58096-8 (PMC11953326; doi:10.1038/s41467-025-58096-8)
Supplement: Supplementary file 1 — Supplementary Information [file 41467_2025_58096_MOESM1_ESM.pdf]

Supplementary Information

Beyond surface tension-dominated water surface jumping

Xin Wang[1†], Neng Xia[1†], Chengfeng Pan[2†]*, Jinsheng Zhao[1], Bo Hao[1], Lin Su[1], Dongdong Jin[3], Qingsong Xu[4], Xurui Liu[1], Xingyu Hou[1], Li Zhang[1,5]*

[1]Department of Mechanical and Automation Engineering, The Chinese University of Hong Kong, Hong Kong, SAR 999077, P.R. China

[2]The State Key Laboratory of Fluid Power and Mechatronic Systems, College of Mechanical Engineering, Zhejiang University, Hangzhou, Zhejiang 310027, P.R. China

[3]School of Materials Science and Engineering, Harbin Institute of Technology (Shenzhen), Guangdong, China.

[4]Department of Electromechanical Engineering, Faculty of Science and Technology, University of Macau, Macau, China

[5]CUHK T Stone Robotics Institute, The Chinese University of Hong Kong, Hong Kong, SAR 999077, P.R. China

*Corresponding Author: cfpan@zju.edu.cn (C.P.); lizhang@cuhk.edu.hk (L.Z.)

†These authors contributed equally to this work.

1. Supplementary Table 1

Supplementary Table 1: Comparison of the jumping performance of the water surface jumping soft robot in the current study with previously reported engineered water surface jumping robot and insects in nature

Mechanism	Body length (mm)	Jumping height (mm)	Jumping height (BL)	Take-off time (ms)	Actuation time (ms)	Take-off velocity (BL/s)	Direction control	Ref.
**This work**	**35**	**630**	**18**	**7**	**0.4**	**100.6**	**Yes**	**This work (soft jumping robot))**
Motor-spring	100	95	0.95	N/A	2	15.70	No	*25*
Latch-spring	15	26	1.73	33	6.9	6	No	*27*
Latch-spring	102	142	1.39	40	15	16.37	No	*1*
Motor-spring	250	140	0.56	N/A	N/A	6.40	No	*22*
Latch-spring	280	545	1.95	45	5	12.86	Yes	*11*
Latch-spring	135	110	0.81	24	12.5	20.74	Yes	*37*
Magnetic field	25	38	1.52	16	16	28	Yes	*34*
**Insects**								
Pygmy mole crickets	5.56	100	18	5.8	1.4	395.68	Yes	*35*
Springtails	0.8	14	16	1.5	0.5	875	Yes	*2*
Water strider (large species)	30	300	10	72	45	53.33	Yes	*10*
Fisher spider	74	37	0.5	92	52	8.11	Yes	*36*
Grasshopper	25	6.75	0.27	43.3	30	20.5	Yes	*37*

2. Supplementary Notes

Note 1: The calculation of the driving force-to-mass ratio (F_{max}/m)

For the high-performance water surface jumping motion, a high driving force-to-body mass ratio for greater acceleration is indispensable. We calculated and compared the F_{max}/m_{total} of the representative water surface jumpers as shown in Supplementary Table 2.

Supplementary Table 2. The comparison of the F_{max}/m_{total} ratio of the representative insects with high performance water surface jumping motion and the soft robot demonstrated in this work.

		Driving force (mN)	Body mass (mg)	F_{max}/m_{total} (N/kg)
Animals	Water strider (1)	6.07	49	1.24×10^{2}
	Springtails (2)	0.038	0.13	2.92×10^{3}
Artificial (Water strider bionic robot) (1)		9.27	68	1.36×10^{2}
This work		5500	410	1.34×10^{4}

For the surface tension-dominated water surface jumping mechanism, the force output of the actuator is strictly limited in order to achieve maximum momentum and avoid breaking the water surface. The actuator's power release is strictly regulated to realize the maximum force output as close as possible to the surface tension of water to obtain a greater driving force and take-off velocity. In contrast, the water surface jumping mechanism proposed in the current work allows the breaking of the water surface, indicating that the actuator release capacity is unrestricted to realize the maximum force output. The larger value compared with the robot based on the water surface dominated mechanism reveals that the current strategy allows increasing the output of the actuator for better performance.

In addition, the total body mass of the jumper is strictly limited by the bearing capacity of water surface in the initial stable float state, necessitating an actuator with a high output driving force-to-body mass ratio ($F_{max}/m_{actuator}$) for greater acceleration. For the data shown in Fig. 3c and 3d, the $F_{max}/m_{actuator}$ ratio is calculated for individual actuation module (i.e., hydrogel actuator and spring actuators) rather than the entire robot system for the comparison. The force measurement of the hydrogel actuator are as follows, the hydrogel actuator is placed on the surface of the force sensor (Nano17ti: acquisition rate is 33.3 kHz)

and triggered by NIR light. The hydrogel actuator hits the force sensor and the instantaneous force data are recorded. For the spring actuators, the maximum force was detected during extreme compression as illustrated in the inset of Fig. 3d and Supplementary Fig. 21. As shown in the Supplementary Table 3, the results show that the hydrogel actuator significantly outperforms spring actuators in terms of the driving force-to-mass ratio and light body.

Supplementary Table 3. The detailed calculation parameters and comparison of the soft spring actuator, hard spring actuator, and our hydrogel actuator.

	Driving force (N)	Body mass (mg)	$F/m_{actuator}$ (N/kg)
Soft spring actuator	6.07	197	3.08×10^4
Hard spring actuator	10.80	356	3.04×10^4
Hydrogel actuator	5.39	110	4.90×10^4

Note 2: Actuation mechanism of the light driven hydrogel actuator

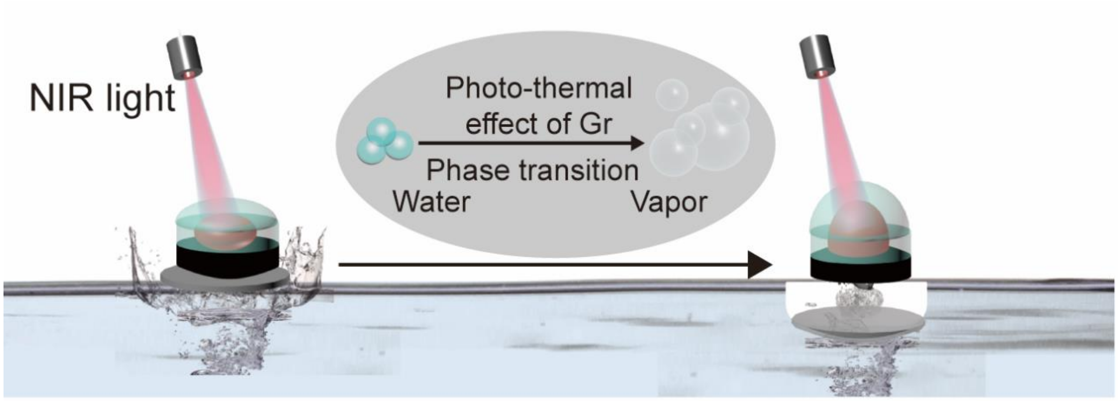

Supplementary Fig. 1. (a) Schematic illustration of the actuation mechanism of the hydrogel actuator.

As shown in Supplementary Fig. 1, the light-driven hydrogel actuator is designed with a double-layer structure to adopt the accumulated strain energy-fracture power-amplification method that reported in our previous work[1]. The bottom layer is composed of a high-toughness hydrogel (Poly(N-vinyl-2-pyrrolidone-co-acrylic acid) (P(VP-co-AA)) hydrogel) embedded with graphene, covered with a pristine hydrogel layer serving as the constraint to control the direction of propulsion. The specific material characterization is illustrated as follows:

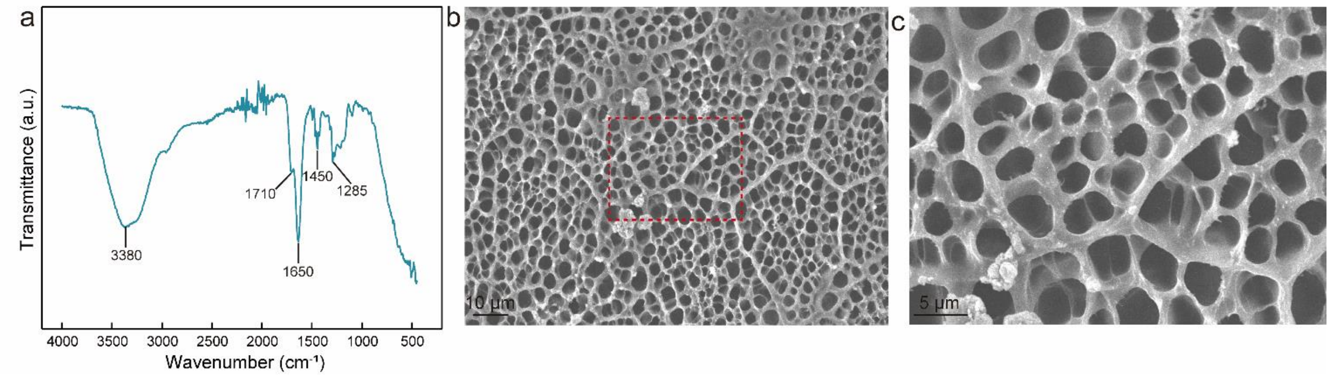

Supplementary Fig. 2. The characterization of hydrogel actuator. (a) The FTIR spectra of the hydrogel actuator. (b) SEM and (c) zoom in images indicating the covalently cross-linked network structure of the hydrogel actuator.

As shown in Supplementary Fig. 2a, the broad absorption band around 3300 cm^{-1} is associated with the C-H group. The characteristic peaks of 1710 cm^{-1} and 1650 cm^{-1} correspond to the C=O stretching band peak in AA and amide on VP, respectively. The peak appearing at 1450 cm^{-1} originates from the stretching band of the pyrrolidone ring, and the peak at 1285 cm^{-1} is the C-N stretching band, the FTIR spectrum proves the copolymer of P(VP-co-AA)[2]. In addition, P(VP-co-AA) hydrogel shows the dense

network structure that endows its mechanical performance as shown in Supplementary Fig. 2b and 2c. We have tested the water absorption ability in water environment (Supplementary Fig. 3 and Fig. 4), the limited volume and weight change within 6 h indicating the excellent anti-swelling properties, which potentially originate from the high-density polymer chains induced by a high degree of cross-linking that limits the entry of water molecules.

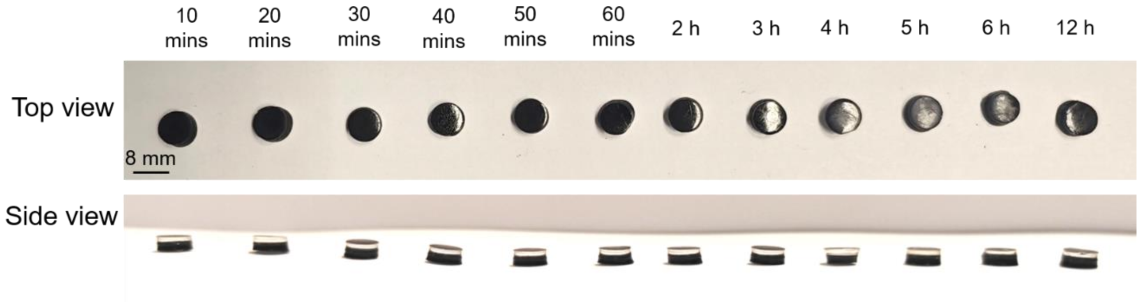

Supplementary Fig. 3. The top view and side view images of the actuators after water absorption over different time periods.

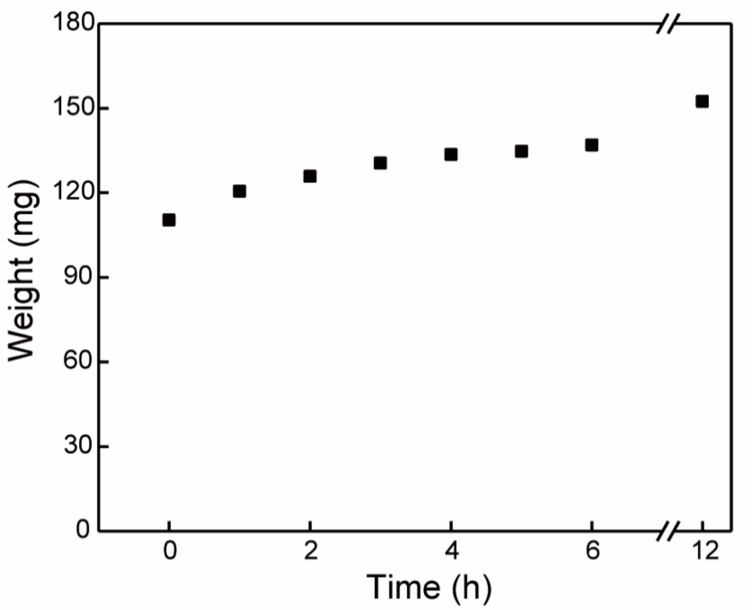

Supplementary Fig. 4. The weight change of the actuator after water absorption over different time periods.

The graphene is doped as photo-thermal agent to produce thermal energy to induce the phase-transition of water. When irradiated with an NIR laser, the photothermal effect of the graphene in the bottom layer produces a large amount of heat that induces dramatic water vaporization inside the hydrogel network, resulting in expansion and strain energy accumulation (Supplementary Fig. 5). Continuous light energy input increases the vapor pressure inside the hydrogel until it reaches a limit, causing mechanical fracture in the bottom surface and instant steam release within 0.6 ms (Supplementary Fig. 6).

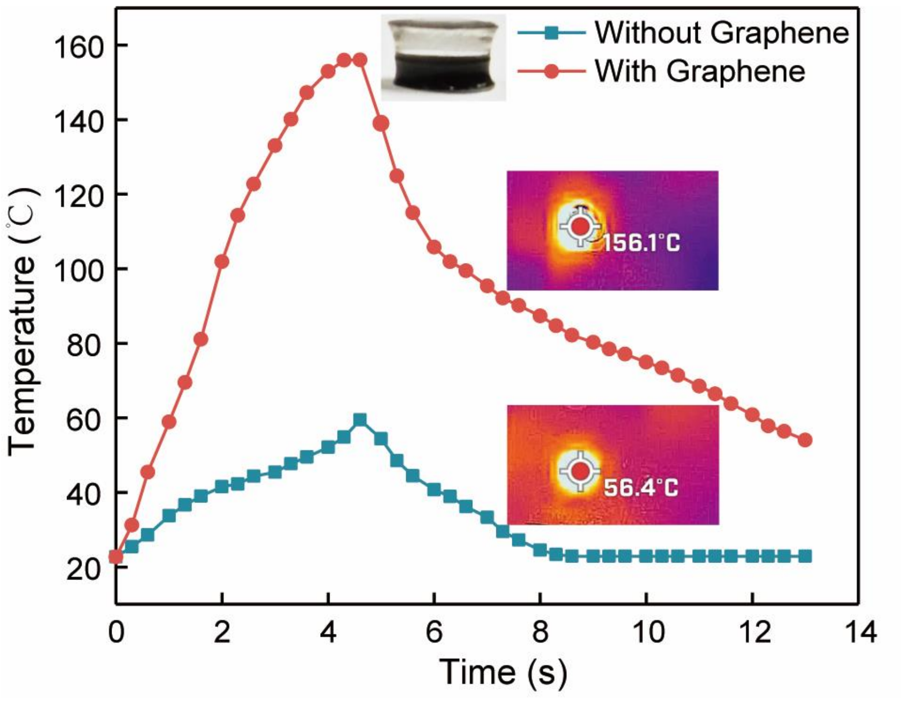

Supplementary Fig. 5. The temperature change of the upper layer (blue curve) and bottom layer (red curve) during the NIR light activating period.

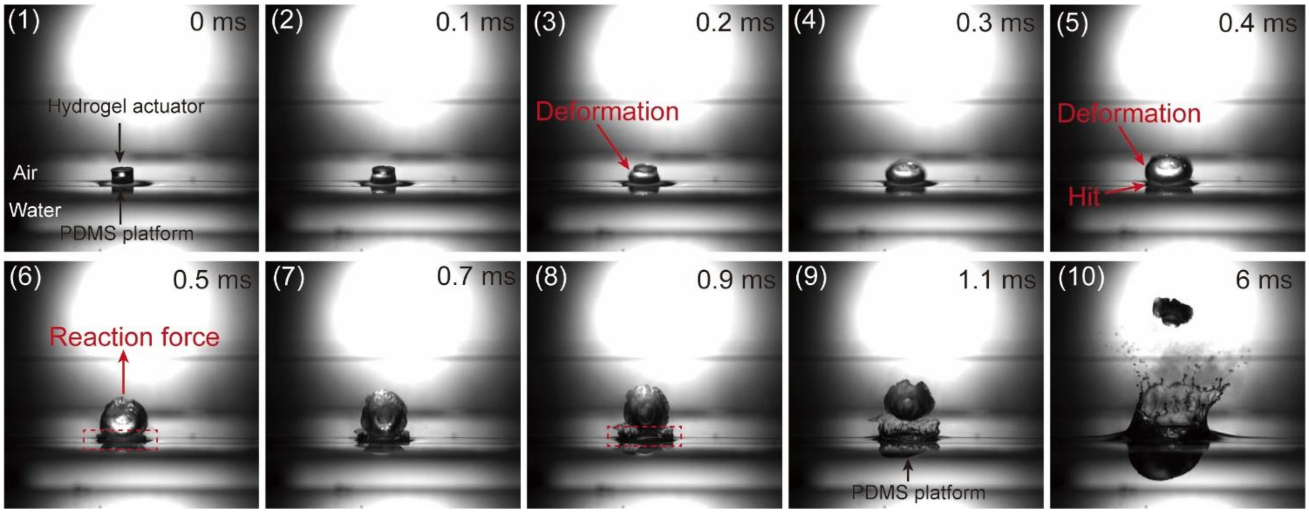

Supplementary Fig. 6. The high-speed camera images showing the take-off process of the hydrogel actuator from the floated PDMS platform on water surface.

Note 3: the mathematical model of the water surface jumping motion

For the process of the jumping motion that after leaving the water surface and reaching the highest point. The drag force is expressed as follows[3]:

$$F_{drag} = C_d \rho A v_f^2/2 \tag{1}$$

Where C_d is the drag coefficient that is strongly determined by shape, ρ is the density of air (1.20 kg/m^3), A is the cross-section area of the jumping robot (Note that the change of the cross-sectional area due to the robot body's rotation during the jump is ignored here, and the cross-section area can be estimated is $A = \pi r^2$ = 50.24 mm^2, and v_f is the body velocity.

The dynamic equation of the jumping behavior can be expressed as:

$$m\frac{d^2x}{dt^2} + \frac{C_d \rho A}{2}\left(\frac{dx^2}{dt}\right) + mg = 0 \tag{2}$$

Where m is the mass (410 mg) of the jumping robot, x is the distance above the water surface, t is the time since the PDMS pad left the water surface, g is the gravitational acceleration, the solution shows the maximum height of the launching process as follows:

$$h = \frac{m}{C_d \rho A} ln\left[\frac{C_d \rho A v^2}{2mg} + 1\right] \tag{3}$$

$$v_{take\text{-}off} = \sqrt{\frac{\left[e^{\frac{hC_d\rho A}{m}} - 1\right] \times 2mg}{C_d \rho A}} \tag{4}$$

Where m is the mass of water surface jumping robot (410 mg) and g is the gravitational acceleration. C_d is the drag coefficient (0.88)[4]. The theoretical take-off velocity calculated from Equation (4) is 3.52 m/s.

The released energy from the explosion of the NIR light driven hydrogel actuator can be calculated as follows:

$$E_{explosion} = E_s + E_b + E_{jumping} \tag{5}$$

Where E_s is the energy loss that overcomes the surface tension, E_b is energy loss during the dynamic interaction with water, and the $E_{jumping}$ is the kinetic energy of the robot.

In the equation (5), the E_s can be calculated through the integral of the water surface during the process of the robot being pulled out of water surface, the experimental setup and the result are shown in Supplementary Fig. 7a and 7b, the E_s can be calculated as follows:

$$E_s = \int_a^b F_s \, dx \tag{6}$$

Where a is the water surface level (set as 0) in the initial state and b is the height when the PDMS pad leaves the water surface, F_s is the surface tension before breaking the water surface, E_s can be calculated from equation (6) and Supplementary Fig. 7b is 0.13 mJ. Note that the work of surface tension is significantly influenced by the pull-off speed, the actual motion velocity is at the m/s level. Due to the limitations of the test equipment, a quasi-static measurement with slow pulling speed (0.1 mm/s) is used to roughly estimate the surface tension. The faster pulling speed tends to result in lower measured surface tension work (i.e., <0.13 mJ), which introduces limited errors in the calculation of energy conversion efficiency compared to the released energy of the actuator (i.e., 5.16 mJ as shown in the end of Supplementary Note 3). Therefore, we use quasi-static test conditions to roughly estimate the work of surface tension.

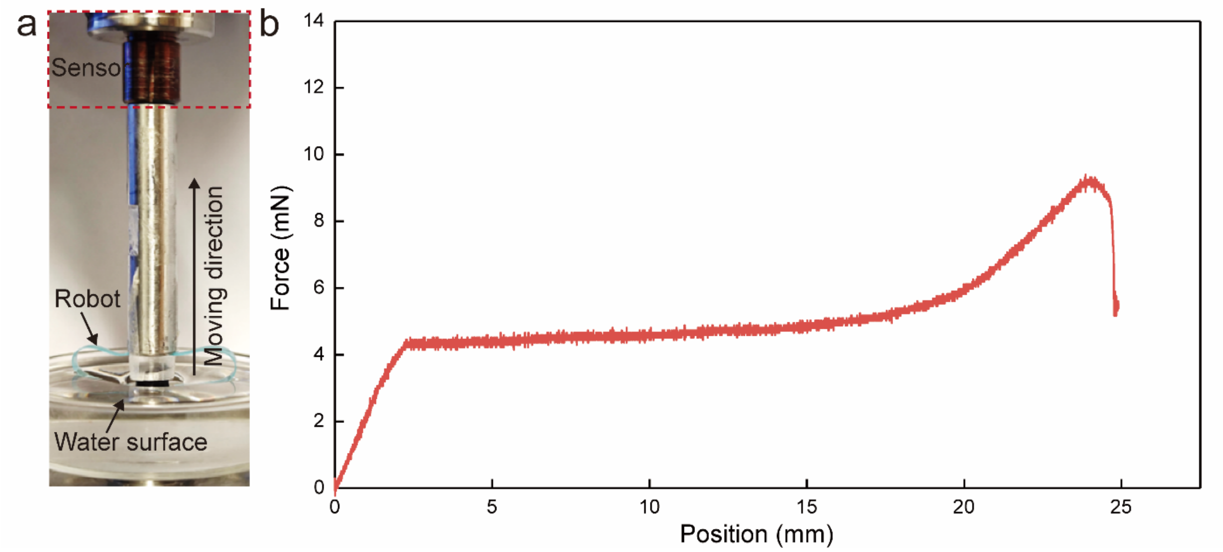

Supplementary Fig. 7. (a) The image of the experimental setup. The robot is mounted on the force sensor and slowly pulled out from the water surface. Note that the pulling speed affects the measured surface tension. Due to the limitations of the test equipment, a slow pulling speed (0.1 mm/s) is used to roughly estimate the surface tension. (b) Force trace as the robot is pulled out of the water surface.

E_b is the energy loss during the dynamic interaction with water, which can be calculated in two parts, i.e., the potential energy and kinetic energy of water displaced beneath the water surface and the splashed water above the water surface, respectively. We analyzed a series of high-speed images and choose the critical frames for calculating (Supplementary Fig. 8), which is the highest position of the splashing water column and the lowest position of the displaced water. At this moment, the kinetic energy is zero and is assumed to have been fully translated to the potential energy of the water.

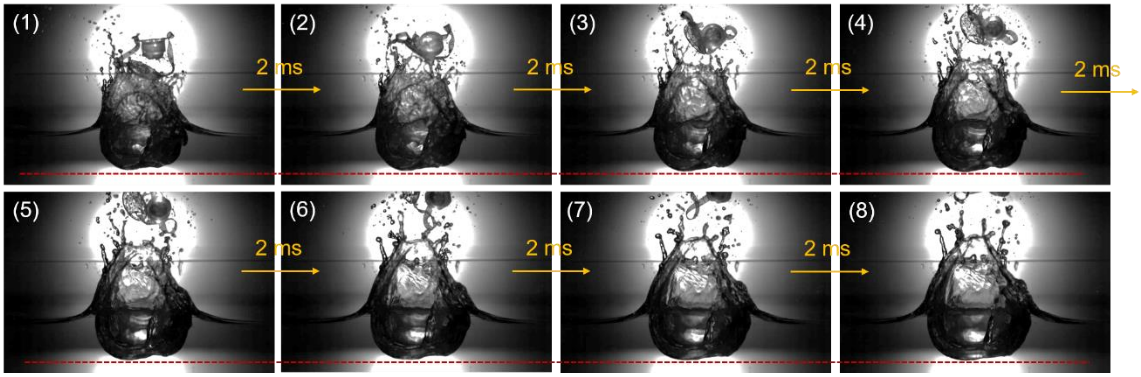

Supplementary Fig. 8. A series of images obtained from Supplementary Movie 1 indicating the moment when the kinetic energy is zero.

Therefore, E_b can be calculated by the maximum potential energy (i.e., kinetic energy is 0) of the displaced water (E_1) and splashed water (E_2) (Supplementary Fig. 9).

$$E_b = E_1 + E_2 \tag{7}$$

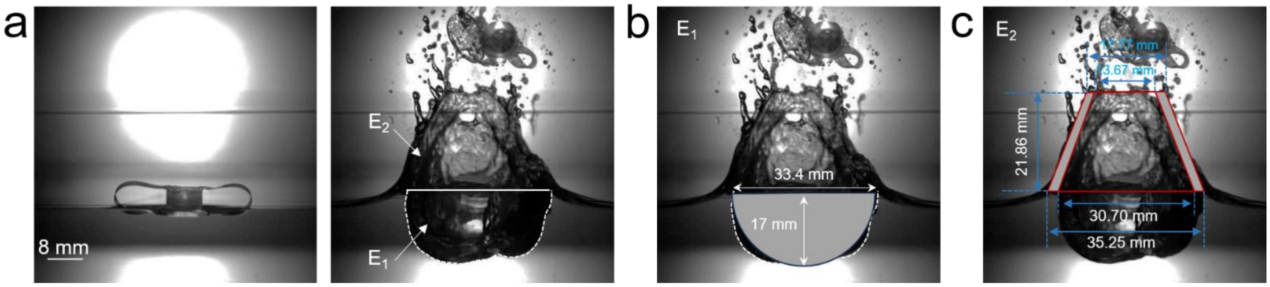

Supplementary Fig. 9. (a) The energy calculation model setting and key frames. (b) The translated calculation model parameters of the maximum potential energy of the displaced water (E_1) and splashed water (E_2).

For the calculation of E_1 that can be calculated through the high-speed image when the displaced water reaches the maximum volume (Supplementary Fig. 9):

$$E_1 = \int_0^{f(x)} F_b \, dx = \int_0^{f(x)} \rho g S dx \tag{8}$$

Where ρ is the density of water, g is the acceleration of gravity, S is the area of the PDMS pad, and $f(x)$ can be identified through high-speed image. In order to simplify the calculation, we use the potential energy of the displaced water as an approximation. The volume of the displaced water can be regarded as a hemisphere that the parameters are shown in (Supplementary Fig. 9), the weight of the displaced water is ~ 10 g and can be calculated as follows,

$$m = \rho V_1 = \rho \cdot \frac{\pi}{3}(3r - h_1) \cdot h_1^2 \qquad (9)$$

Where ρ is the density of water, r is the radius of the hemisphere, and h_1 is the distance between the water surface and the lowest position of the cavity.

Therefore, the energy can be roughly estimated as follow,

$$E_1 = \int_0^{f(x)} F_b \, dx \approx m_1 g h_1 \qquad (10)$$

The E_1 calculated is 1.7 mJ.

Similarly, as shown in Supplementary Fig. 9, the volume of the splashing water column can be roughly estimated by considering it as a hollow truncated cone, the thickness of the truncated cone can be estimated by the transparency distribution in high-speed camera screenshots, the weight of the splashed water is ~ 3.63 g can be calculated as follows,

$$m_2 = \rho(V_{outer} - V_{inner}) \qquad (11)$$

Where ρ is the density of water, V_{outer} and V_{inner} are the volume of the outer and inner truncated cone, respectively, which can be calculated by $V = \frac{1}{3}\pi h(R^2 + Rr + r^2)$ based on the parameters given in Supplementary Fig. 9.

Therefore, the potential energy of the splashed water above the water surface is 0.79 mJ that can be roughly estimated by,

$$E_2 = m_2 g h_2 \qquad (12)$$

The energy loss during the dynamic interaction with water is calculated to be 2.49 mJ.

The $E_{jumping}$ can be calculated based on the Newtonian equation as:

$$E_{jumping} = \frac{1}{2} m v_{take-off}^2 \qquad (13)$$

Where the $v_{take\text{-}off}$ is the body velocity of the robot. This can be determined by equations (1 - 4) based on the recorded jumping height. In addition, it can also be analyzed by the momentum theorem at the moment when the tendon is straightened:

$$v_{take-off} = \frac{m_b v_b + m_p v_p}{m_b + m_p} \tag{14}$$

Where m_p and v_p are the mass and the velocity of the PDMS pad, respectively. m_b and v_b are the mass and the velocity of the robot's body (i.e., the actuation module), respectively. When the explosion occurs, the PDMS pad and the hydrogel actuator loaded actuation module experience the same amount of force in opposite directions. For the PDMS pad, the downward velocity attenuates rapidly because it experiences the resistance force including buoyancy, hydrodynamic force, and the surface tension of water during the downward movement. In comparison, the actuation module only experiences gravity and air resistance, which shows limited velocity reduction. The greater the driving force provided, the greater the velocity difference between the PDMS pad and the actuation module at the moment when the tendon is straightened, resulting in a whole upward movement with greater take-off velocity (equation (14)) and better jumping performance. This is the reason why the current split-type structure design of the soft robot allows the full release of the stored energy of the actuator. The kinetic energy is estimated to be 2.54 mJ.

Therefore, the released energy applied to the PDMS pad of the hydrogel actuator for the jumping actuation can be expressed as:

$$E_{explosion} = E_s + E_b + E_{jumping} \tag{15}$$

Based on this, the released energy calculated through equation (15) is 5.16 mJ. And the energy conversion efficiency can be calculated through equation (16) is 49.22 %.

$$\eta = \frac{E_{jumping}}{E_{explosion}} \tag{16}$$

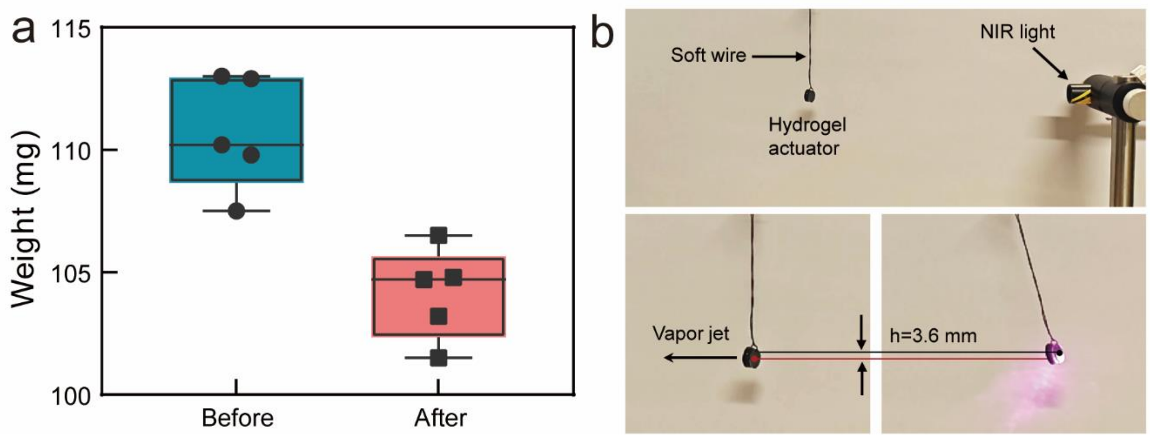

Supplementary Fig. 10. (a) The weight difference before and after the light driven launching motion. Data points are shown as mean ± s.d. (n = 5). (b) The vertical displacement of the hydrogel actuator's center of mass which actuated by the vapor jet.

The phase-change of water induces the deformation and strain energy accumulation, we have measured the average weight loss (i.e., water loss before and after the actuation) is 6.54 mg for each actuation, such a small amount of water vapor evaporation indicates the main source of actuation for high-speed motion is not the vapour jet propulsion (as shown in Supplementary Fig. 10a). We have roughly calculated the energy lost from the vapour jet. Specifically, as shown in Supplementary Fig. 10b, the hydrogel actuator is suspended in the air by a soft and thin wire, the vapour jetting can induce the pendulum motion of the hydrogel actuator in air. The energy production from the vapour jetting can be roughly calculated from the increase of the gravitational potential energy (U_g):

$$U_{vapour} = U_g = mgh \tag{17}$$

Where m is the mass of the hydrogel actuator, h is the vertical displacement of the center of mass as shown in Supplementary Fig. 10b, the calculated result of 3.96×10^{-3} mJ is negligible compared to the kinetic energy of the robot ($E_{jumping}$ = 2.54 mJ) and released energy of hydrogel actuator ($E_{explosion}$ = 5.16 mJ), respectively.

Note 4: The optimization of the hydrogel actuator

The characteristics of the actuation module are the fast-response and large driving force to generate high acceleration and initial velocity, thereby improving the jumping performance based on the proposed mechanism. In this work, we adopted our previously reported hydrogel actuator published in Nature Materials with further optimizations for the actuation of water surface jumper as a demonstration.[1] The optimizations in terms of structure design, adjustment of the material's mechanical properties, and trigger operation are detailed as follows,

(1) Structural design. As shown in Supplementary Fig. 11, for the hydrogel launcher demonstrated in the paper published in Nature Materials, the graphene nanoparticles are evenly dispersed throughout the launcher's body. The control of the phase-transition volume inside the hydrogel launcher is based on the light blocking effect of graphene (i.e., light incident from the launcher's surface can only penetrate to a certain depth), and the self-launching motion can only be achieved by placing the hydrogel launcher on transparent substrates. Specifically, when light is irradiated from the bottom that it can only penetrate to a limited depth, causing heat to be generated inside the hydrogel launcher closer to the bottom surface. The strain energy accumulated during the phase change that induce the deformation process, which eventually triggers the explosion in the downward direction to acquire the actuation force. However, for the actuation of the water surface jumping soft robot, the water surface environment restricts the light incident from the air into the water. We solved this problem by changing the structural design to ensure that the phase change region remains inside the actuator close to the bottom surface. As shown in Supplementary Fig. 11, we adopt a two-layer structural design with a transparent hydrogel layer fabricated that allows the light to be incident from the upper surface while keeping the phase-change volume close to the bottom surface inside the actuator. In other words, it is possible to control the explosion position and force output directions based on the multi-layer strategy, i.e., the black phase-change layer and the transparent constraint layer.

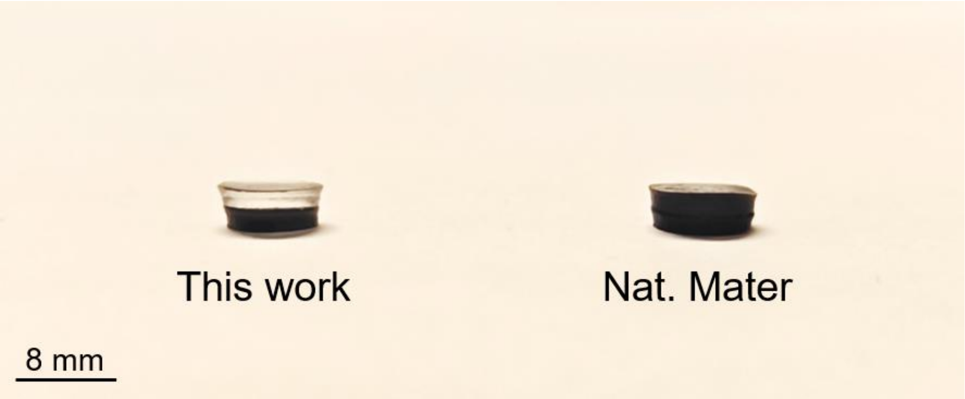

Supplementary Fig. 11. The structural differences of the hydrogel actuators demonstrated in this work and Nature Materials.

(2) Optimization of materials and mechanical properties. The basic principle of the power amplification is long-term energy accumulation and instant release. The greater the energy accumulation and the faster the release, the better power amplification and force output. Compared to the material system (i.e., P(AM-co-AA) hydrogel) of the hydrogel launcher demonstrated in Nature Materials, we have fabricated a similar copolymerized hydrogel system of P(AA-co-VP), which has a higher Young's modulus, as shown in the Supplementary Fig. 12a, indicating the higher strength of hydrogel matrix. Based on the latch-spring mechanism, an increase in the latch strength results in more energy accumulation and faster energy release, thereby inducing the better power amplification effect and greater force output.[5] We have compared the F_{max}/m ratio of the two hydrogel actuators as shown in Supplementary Fig. 12b, the hydrogel actuator illustrated in this work has a larger F_{max}/m output value. Note that the forces are measured directly on the force sensor.

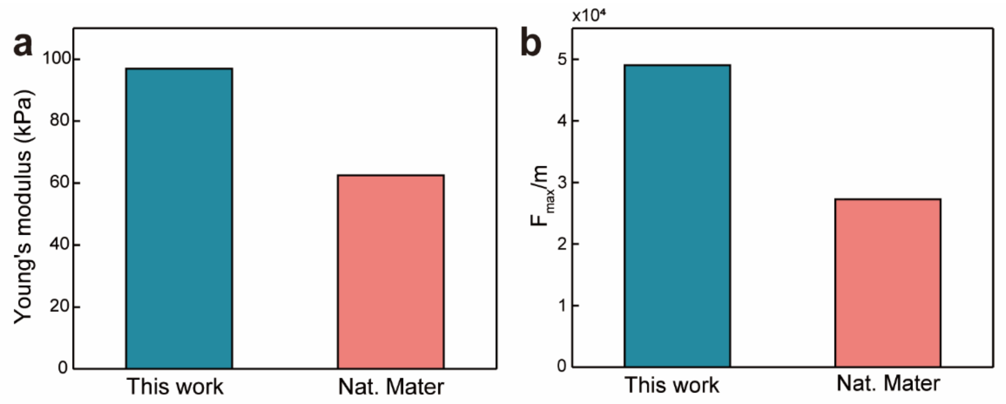

Supplementary Fig. 12. (a) The Young's modulus and (b) F_{max}/m ratio comparison of the hydrogel actuators demonstrated in this work and Nature Materials.

(3) Phase transition volume. The phase transition volume was increased by changing the spot area of NIR light irradiation. As shown in Supplementary Fig. 13a, the larger phase transition volume achieved by increasing the light irradiation area can induce more energy accumulation before fracture, thus leading to greater force output. i.e., increase of the F_{max}/m ratio. The water loss of the hydrogel actuator after actuation in this work is higher than the launcher illustrated in Nature Materials (6.54 mg *vs* 3.20 mg), proving more vapour jetting induced by phase transition and more energy production for transfer and transformation. In addition, we have compared microscope images of the sectional view after actuation (Supplementary Fig. 13b), the phase transition volume and rift of the hydrogel actuator in this work are much larger than the actuator demonstrated in the paper published in Nature Materials, indicating more intense energy production and output.

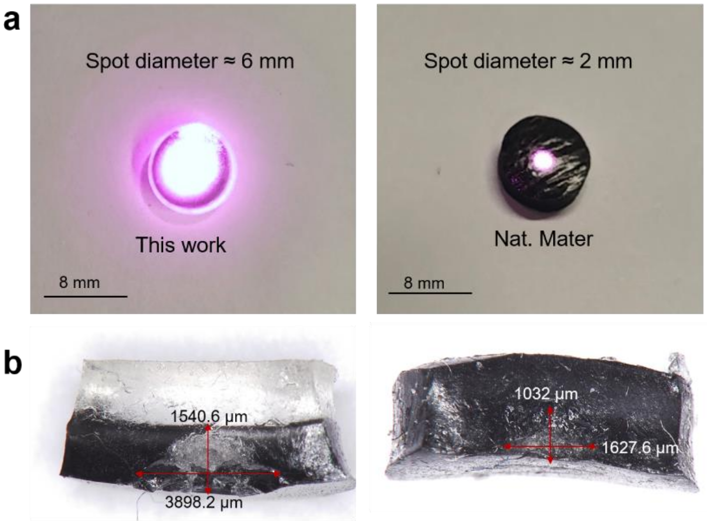

Supplementary Fig. 13. (a) The spot area and (b) phase-transition volume comparison of the hydrogel actuators demonstrated in this work and Nature Materials.

The optimization of the hydrogel actuator aims to achieve faster energy release and a higher *F/m* ratio to meet the requirements of the actuation module for the high-performance water-jumping strategy we proposed. The focus of this work is to break through the limitation of the surface tension dominated mechanism to realize high-performance water surface jumping robot. The surface tension dominated water surface jumping mechanism faces an inherent physical constraint: the propulsion force must remain below the threshold required to break the water surface (i.e., to avoid breaking the water surface). Our design strategy allows the water surface to be broken due that the actuation force originates from the solid interaction rather than the reaction force from the water surface. Each module in our design (i.e., hydrogel actuator, superhydrophobic pad, soft tendons) serves as the representative example help to illustrate the mechanism, and we also described their characteristics. For the hydrogel actuator, which is just an example that we found conveniently and that meets the above characteristics. We clarify that any rigid or soft actuator with the above characteristics can be applied to the actuation of high-performance water surface jumping robots based on the proposed principle.

Note 5: The influence factors of the jumping performance of the soft robot.

For the current design of the soft robot, the jumping performance is influenced by the energy input (light intensity), energy production (graphene content), force output of the hydrogel actuator (mechanical properties of the hydrogel actuator) and the properties of the launching pad and soft tendon. As shown in Supplementary Fig. 14a, the increased light intensity means faster thermal production and deeper penetration, which induces the lagger phase transition volume inside the hydrogel actuator and more vapor accumulation before fracture, thus increasing the force output of the hydrogel actuator, i.e., the more energy input, the more energy transformation and output. The composition of the hydrogel actuator influences the force output of the actuation module. Specifically, the transformed thermal energy to trigger the phase transition originates from the photothermal effect of the graphene, the jumping performance increased first as the graphene content increased from 0.07 wt% to 0.17 wt%, the more and faster thermal production and higher intensity steam explosion enabled by the increased graphene content induce better jumping performance (Supplementary Fig. 14b). However, the excess graphene content (i.e., the graphene content more than 0.17 wt%) leads to the decreased penetration depth of NIR light due to the blocking effect, which induces the smaller phase transition volume (Supplementary Fig. 14c) and lower force output exhibited as the reduction of the jumping height. The concentration of the graphene content needs to be carefully selected in order to balance the trade-off between the photothermal energy conversion efficiency and phase transition volume.

The mechanical property of the hydrogel also affects the force output by changing the energy accumulation ability before fracture. The more strain energy accumulated through mechanical property design during the deformation process (i.e., phase transition inside the actuator before fracture), the greater force output at the moment of fracture and the better actuation performance. Specifically, the water content and crosslinker (MBA) content were changed to tailor the properties of the hydrogel actuator. Supplementary Fig. 14d and the representative stress-strain curve in Supplementary Fig. 14e show that the water content through influencing the toughness to change the energy storage before hydrogel fracture, the difference in energy storage ability results in the exhibited tendency of jumping performance (i.e., first increase and then decrease), the best jumping performance is observed in the water content of 60 wt% means that the biggest energy accumulation before fracture. The effect of the crosslinker content on the jumping performance exhibits the similar trend, as presented in Supplementary Fig. 14f and Fig. 14g, the best jumping performance is realized of the hydrogel actuator concludes the MBA content is 0.23 wt%.

In addition to the influencing factors from the force output of the hydrogel actuator, the superhydrophobic structure of the PDMS pad and the properties of the tendon can also affect the jumping performance. We have measured the influence of the PDMS pad's diameter on the jumping performance of the soft robot, the larger contact area induces the greater surface tension which means the stronger resistance (the diameter ranges from 7 mm to 13 mm), in other words, the soft robot can suffer a greater force output from the actuator before sinking, which leads to a greater velocity difference between the PDMS pad and actuation module to obtain a higher take-off velocity. However, the excess contact area (i.e., > 13 mm) increases the gravity of the soft robot and the resistance (i.e., surface tension as resistance when lifting) during the jumping process, which induces the rapid degradation of the jumping performance (Supplementary Fig. 14h). Moreover, the soft tendon is indispensable for high performance water surface jumping motion. The jumping robot without soft tendon or with rigid material connections cannot realize high-efficient momentum transfer inside the robot's body, resulting in unsuccessful jumping or low jumping performance. The mechanical properties of the soft PDMS tendon (i.e., the length and softness) also influence the water surface jumping performance. We performed the vertical water surface jumping motion of the soft robot with tendons of different stiffness, which fabricated by changing the ratio of the monomers and the hardeners of the PDMS (Supplementary Fig. 14i). The stiffness of the tendon should be chosen carefully because it provides different reaction force ratios for impact.[6] For example, the tendons that is too soft fabricated with Ecoflex will increase energy dissipation due to large deformation during the stretching process. The optimal tendon stiffness is fabricated with PDMS-10 that can achieve the best jumping performance. Furthermore, the jumping performance is also influenced by the length of the soft tendon as shown in Supplementary Fig. 14j. The jumping performance shows limited differences for the water surface jumping soft robot with the tendon length less than 3 cm, and it decreases rapidly as the length continues to increase. Potential reason is that the actuation module of the soft robot moves upward due to the reaction force generated by the fractured hydrogel actuator hitting the PDMS pad, resulting in the initial acceleration caused by the instant impulse and subsequent deceleration motion. The length of the tendon should match the displacement distance of the acceleration stage to obtain the best jumping performance.

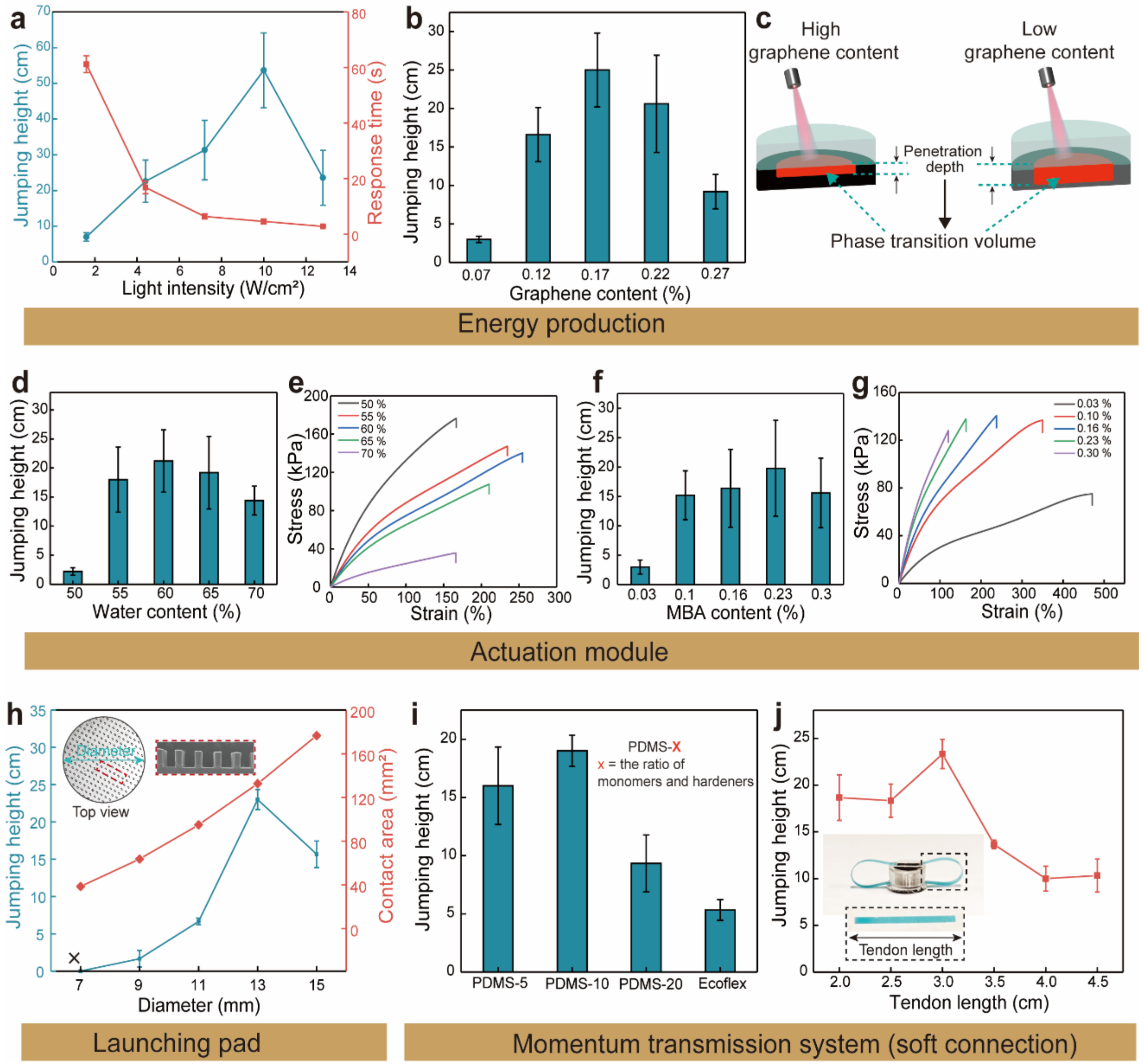

Supplementary Fig. 14. The influencing factors of the jumping performance of the soft robot. (a) The jumping height and response time of water surface jumping motion for soft robot irradiated with different light intensity. (b) The jumping height of the water surface jumping motion for soft robot with different graphene content. (c) Schematic illustration of the graphene content induced the difference of phase transition volume inside the actuator's body. (d) The jumping height of water surface jumping motion for soft robot and stress-strain curve of hydrogel with different (d, e) water content and (f, g) MBA content, respectively. (h) The jumping height and contact area with different diameters of pad. (i) The jumping height of the soft robot with different (i) softness and (j) length of the tendon. Note that the data are captured with the light intensity is 2.8 W (4.4 W/cm^2).

Note 6: Summary of the proposed water surface jumping mechanism

Currently, the reported miniature water surface jumping robots mainly rely on surface tension to provide actuation force for water surface jumping motion. However, this mechanism faces an inherent physical constraint: the propulsion force must remain below the threshold required to break the water surface (144 mN/m). The power output of the actuator is strictly controlled in order to avoid the momentum acquisition reduction induced by water surface broken.[7] In other words, powerful actuators with considerable force output have been developed, but the force output ability is constrained during the application by this mechanism. Our design strategy allows the force output ability of the actuator to be released without considering the water surface broken due that the actuation force originates from the solid interaction rather than the reaction force from the water surface. Previously reported water surface jumping robots are basically fabricated with rigid materials and an integrated structural design. This design principle tightly combines the power output and energy conduction with the dynamic water interactions. The limitation brought by the surface tension of water must be considered if we want to improve the power of the actuator for better jumping performance.

For the current design principle, we pioneered the use of soft materials to build water surface jumping robots. Despite the simple structural design (i.e., soft tendons connection), the initial momentum acquisition and the interaction between the robot and the water surface are separated. The actuation force mainly comes from the solid interaction (the actuator hits the launching pad to generate reaction force) rather than the reaction force from the water surface. This actuation force acquisition strategy allows increase the power output of the actuator without considering the limitation of the water surface broken, which maximizes the initial velocity while minimizing the kinetic energy loss due to the water surface broken.

In particular, we clarify that the proposed strategy inspired by biological models is a universal mechanism that can be achieved in different water surface robot designs. We outline three important design principles for achieving high-performance water surface jumping in this work, i.e., Superhydrophobic contact with water surface, momentum transmission system achieved by split structure design, and light weight, powerful actuation module with high *F/m* ratio (Supplementary Fig. 15). The soft robot demonstrated in this work is only a typical example that satisfies the above-mentioned requirement. Any further designs that align with the proposed strategy can be utilized to realize the high-performance water surface jumping motion.

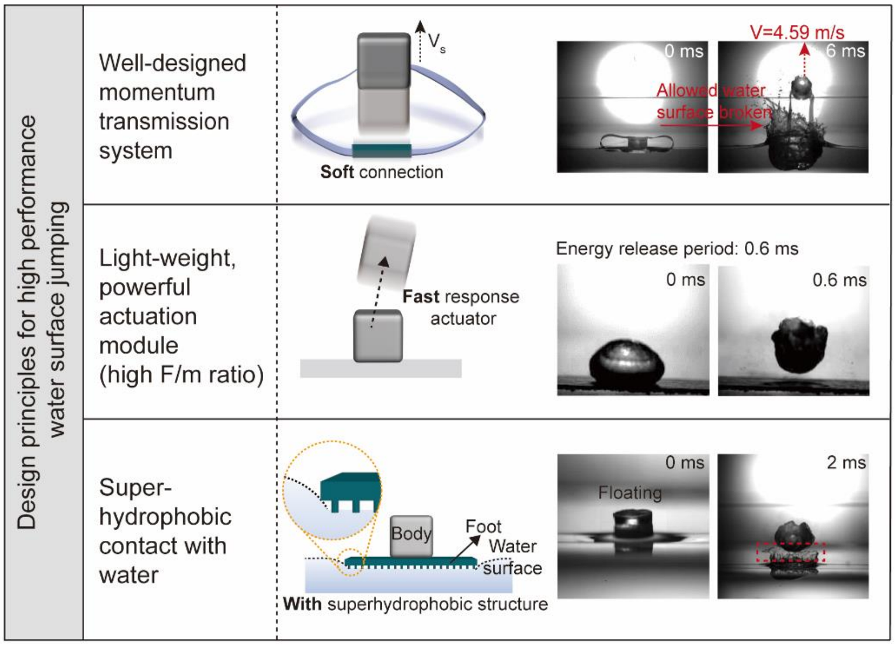

Supplementary Fig. 15. The key design principles of the proposed water surface jumping mechanism.

3. Supplementary Figures

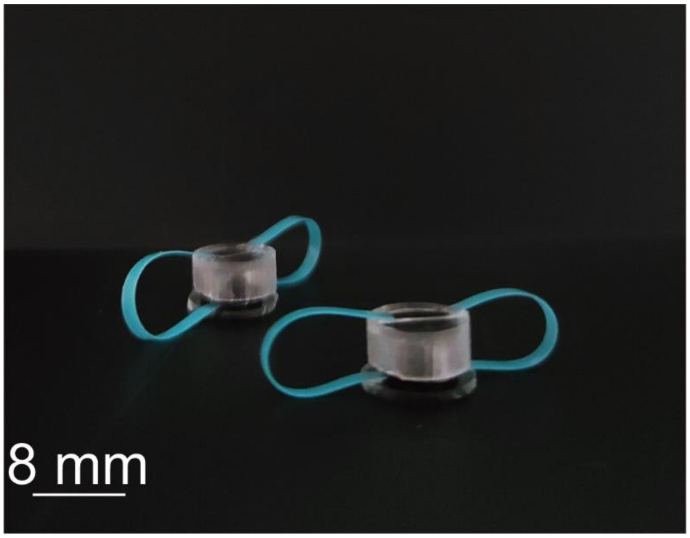

Supplementary Fig. 16. The image of the demonstrated soft robot based on the proposed design principles.

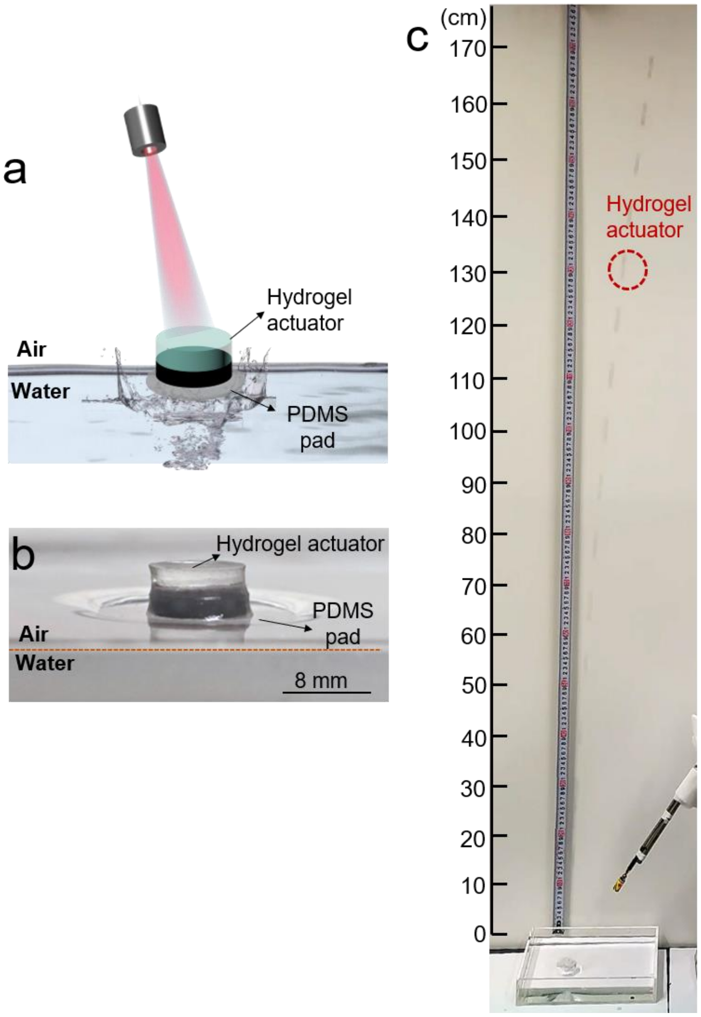

Supplementary Fig. 17. (a) Schematic illustration and (b) image of the initial state for jumping motion of the hydrogel actuator without the remove the launching pad from water. (c) The overlaid image shows the launching height of the hydrogel actuator.

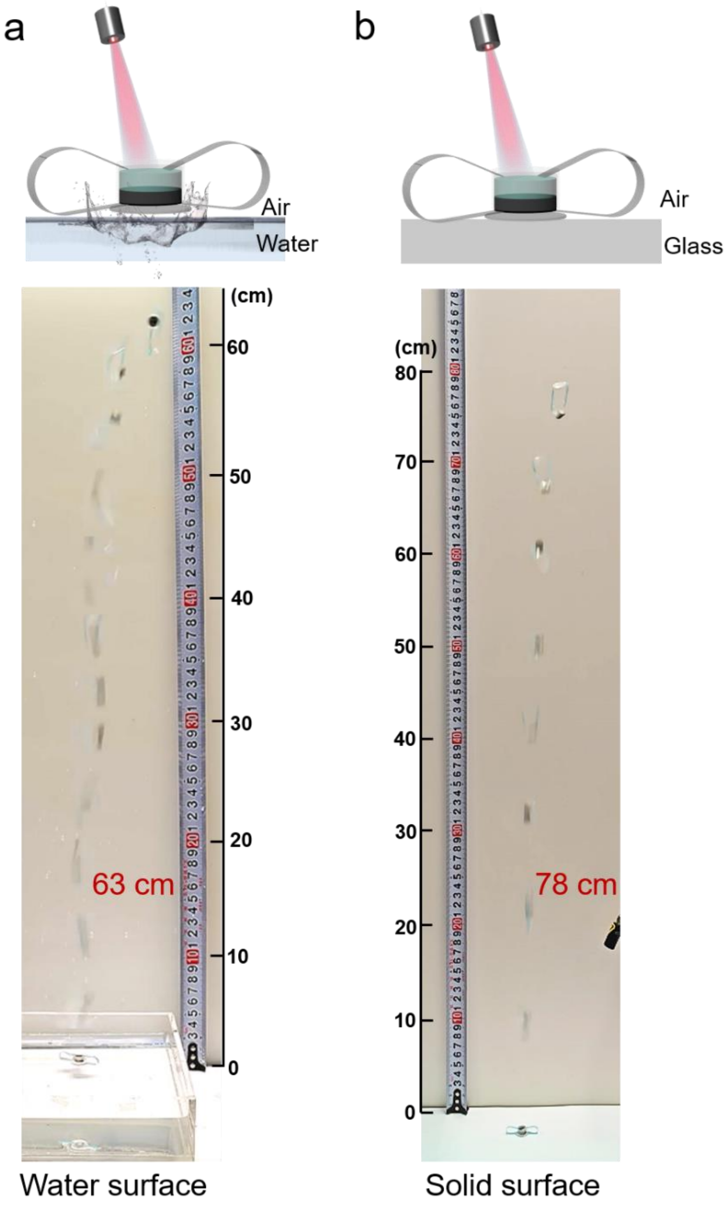

Supplementary Fig. 18. The overlaid image obtained from Supplementary Movie 2 indicating (a) the water surface jumping height and (b) the solid surface jumping height of the soft robot.

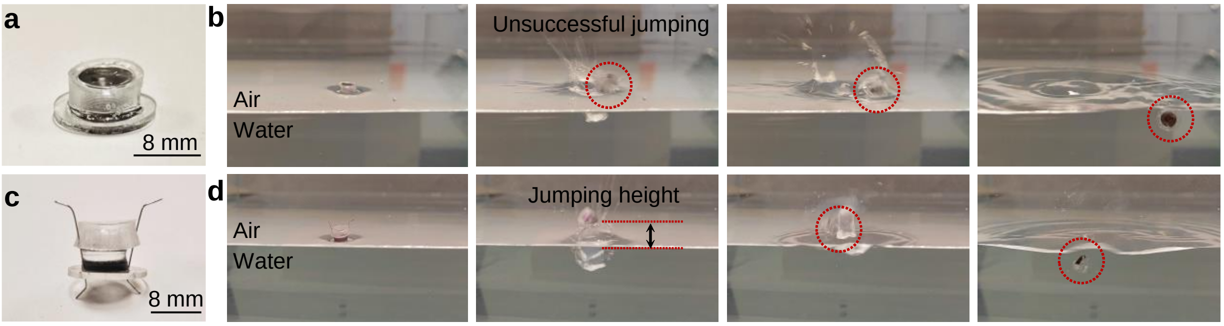

Supplementary Fig. 19. (a) Photograph of the robot use direct connection method and (b) series of images illustrating the corresponding unsuccessful water surface jumping motion. (c) Photograph of the robot with rigid connection method and (d) series of images illustrating the corresponding low performance water surface jumping motion.

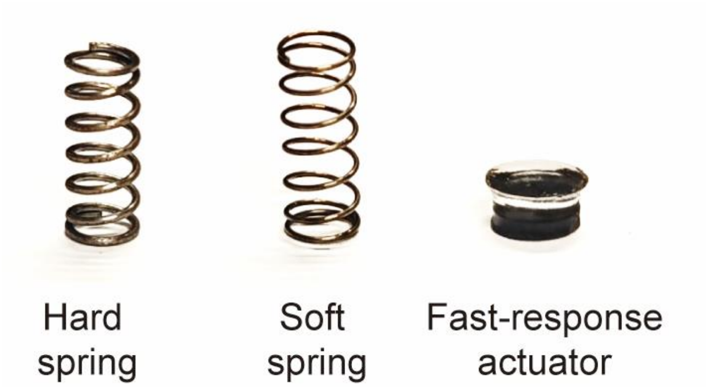

Supplementary Fig. 20. The image of the different actuators used in Fig. 3c.

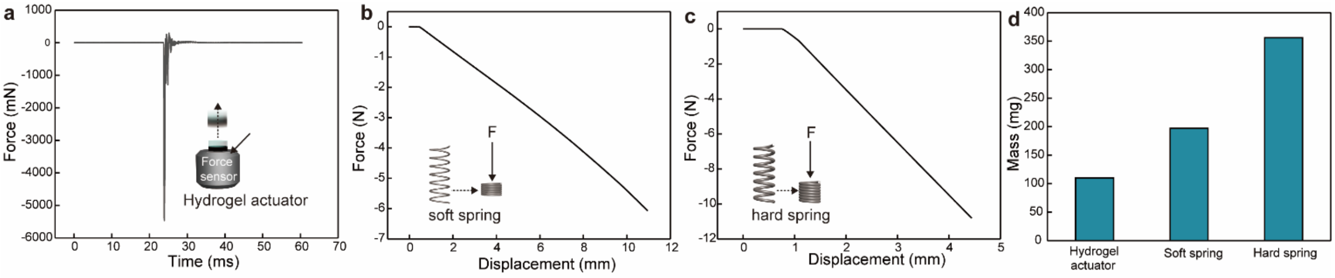

Supplementary Fig. 21. The force measurement of the (a) hydrogel actuator, (b) hard spring and (c) soft spring. (d) The mass comparison of the three actuators.

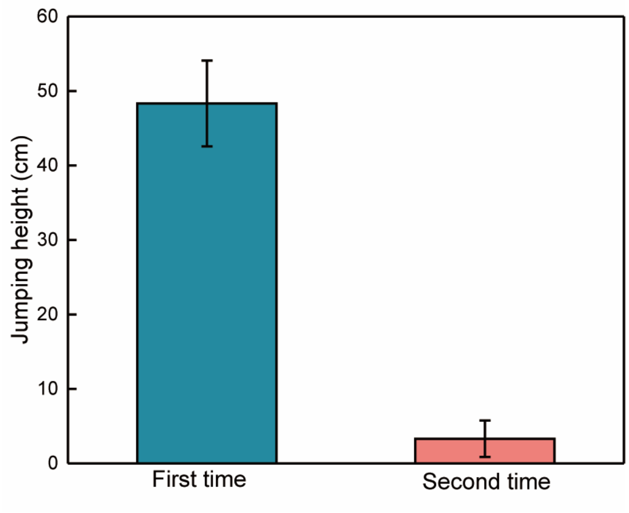

Supplementary Fig. 22. The water surface jumping height comparison of the first and second actuation of the soft robot.

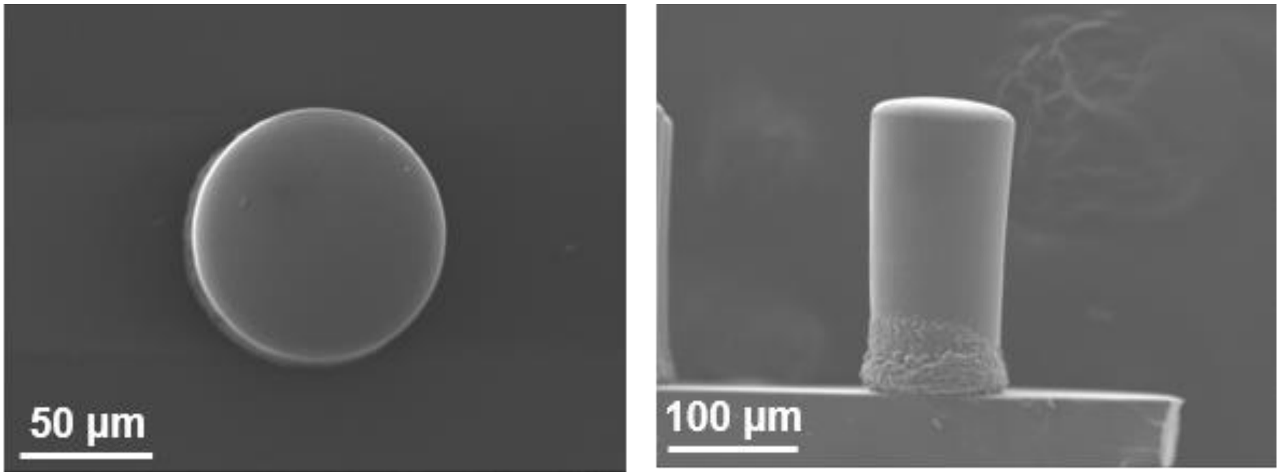

Supplementary Fig. 23. The SEM image of the top view and side view of the micropillar on the surface of the PDMS pad.

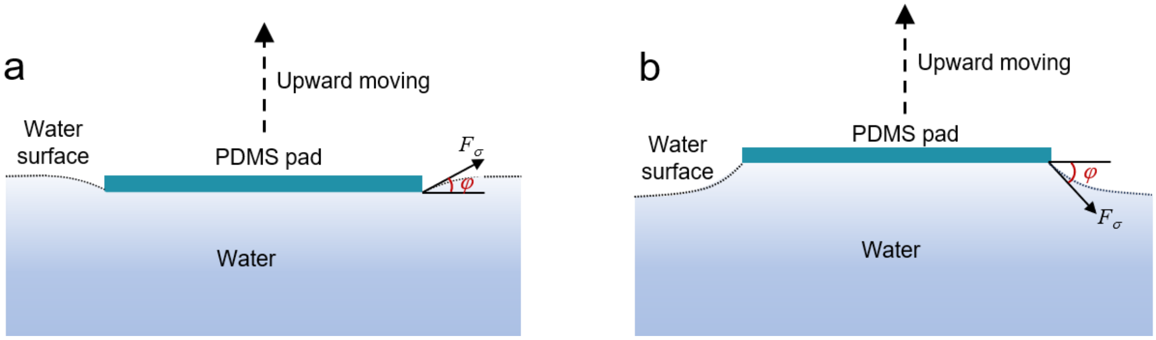

Supplementary Fig. 24. Schematic illustration of the change of the surface tension and the angle of the air-water interface during the PDMS pad upward moving process.

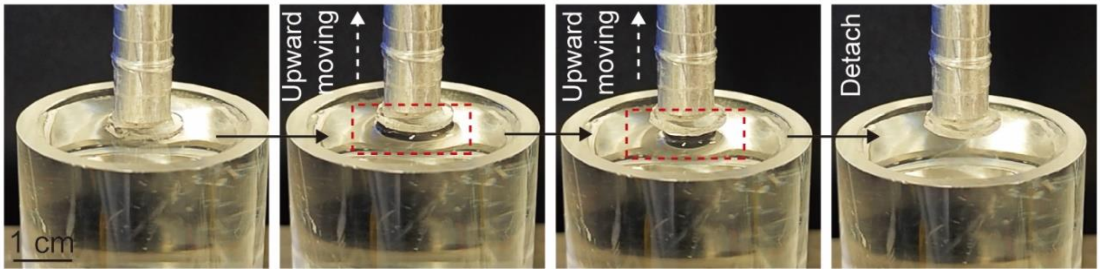

Supplementary Fig. 25. The experimental snapshots illustrate the liquid surface change during the process of the PDMS pad pulled out from water surface. Note the diameter of the pad is 1 cm.

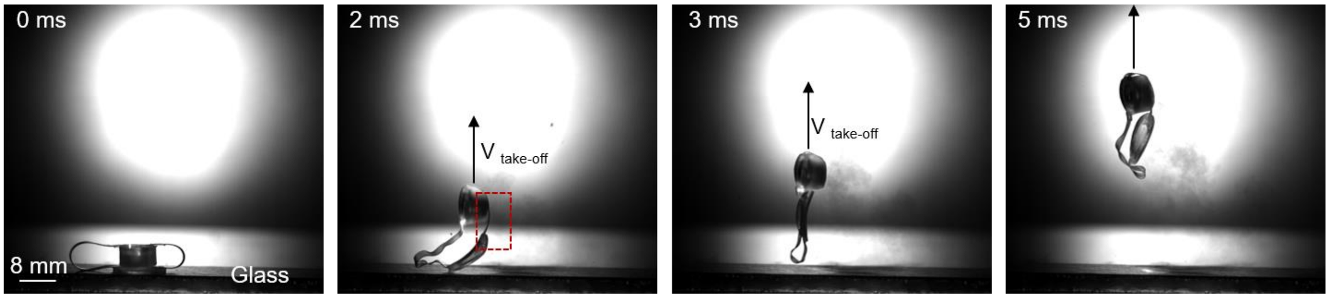

Supplementary Fig. 26. The high speed camera images showing the jumping motion from the glass surface of the soft robot. Note that the left/right tendon length ratio is 3 : 1.5 (cm).

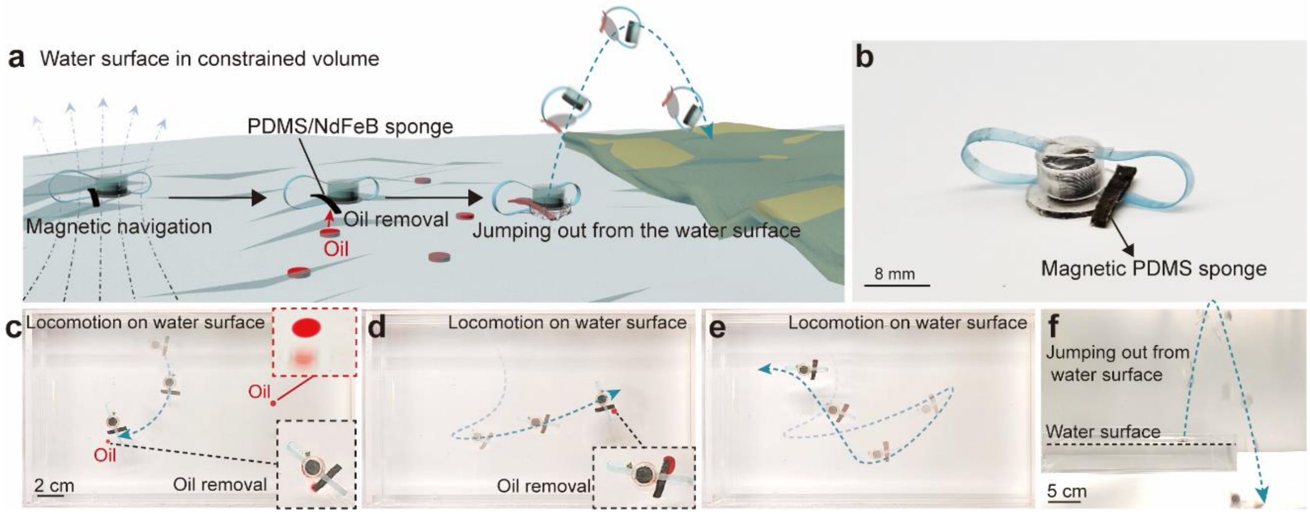

Supplementary Fig. 27. (a) Schematic illustration of the water surface jumping soft robot combined with a magnetic navigation module to realize the water surface oily pollutants removal in a constrained volume. (b) The image of the water surface jumping soft robot integrated with a magnetic oily pollutants absorption module. (c-f) A series of images illustrates the oily pollutants absorption process navigated by magnetic field and followed oil removal.

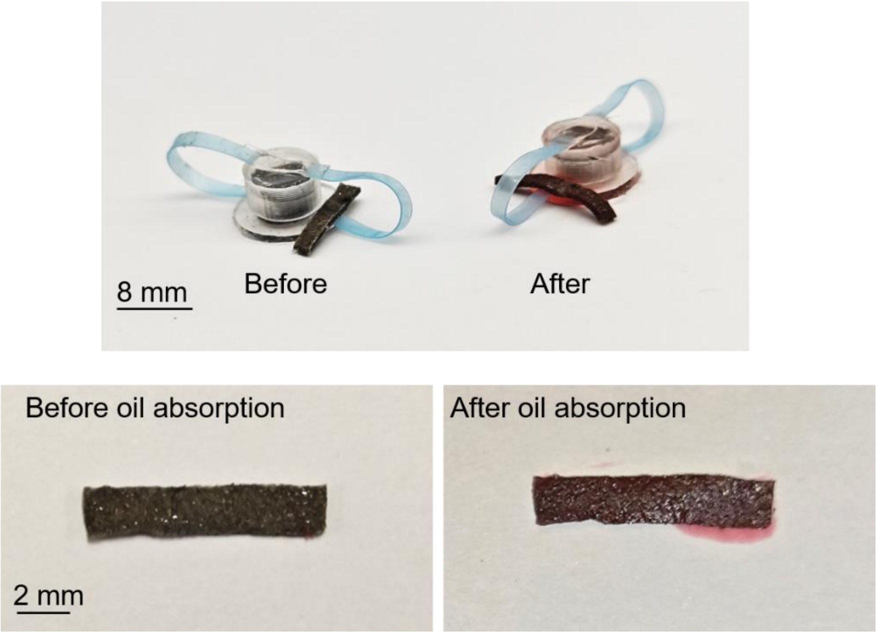

Supplementary Fig. 28. The images of the soft robot and magnetic sponge before and after the oily pollutant absorption.

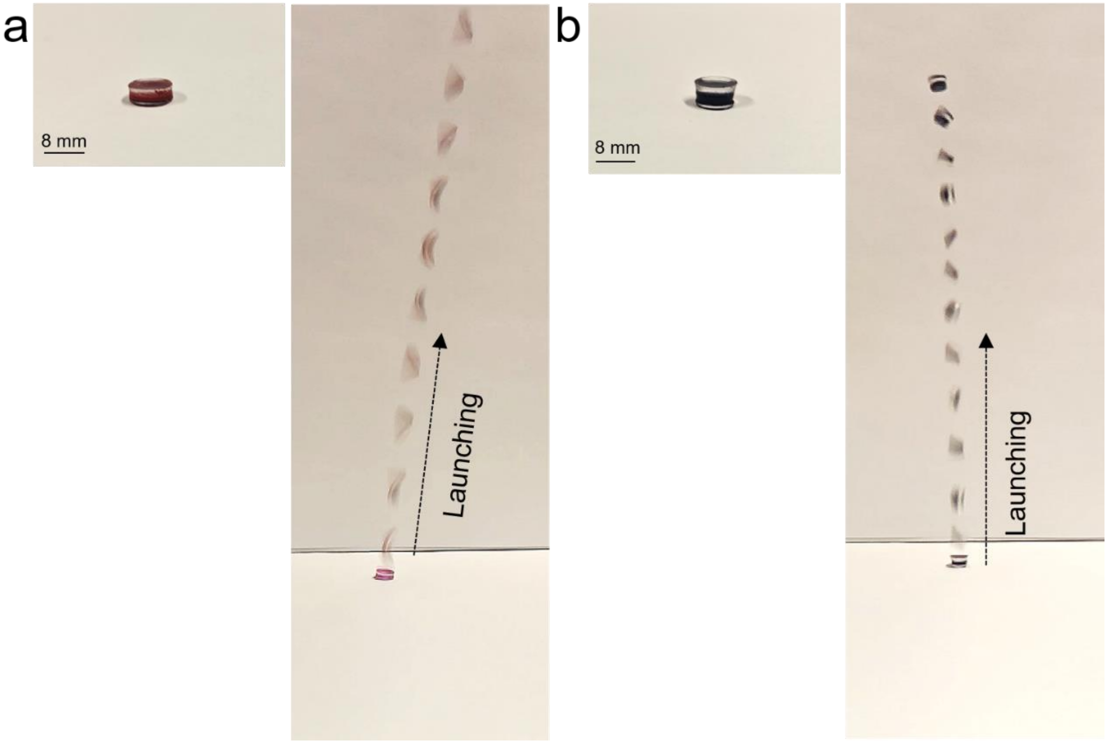

Supplementary Fig. 29. (a) The images and overlaid motion images of the actuators fabricated by doping (a) the Fe_2O_3 nanoparticles and (b) black ink in the hydrogel network.

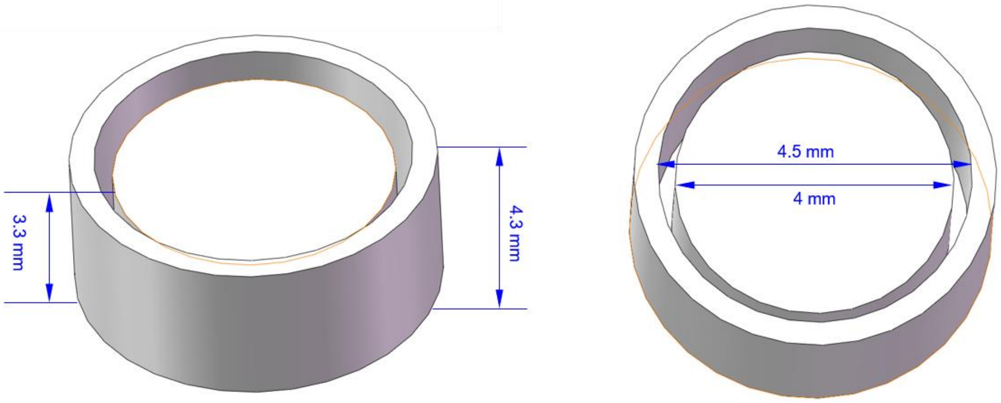

Supplementary Fig. 30. The parameters of the mold to fabricate the actuator-loaded PDMS module.

3. Supplementary Reference

1. Wang, X. et al. Fracture-driven power amplification in a hydrogel launcher. *Nat. Mater.* **23**, 1428-1435 (2024).
2. Kizilbey, K., Derman, S. & Mustafaeva, Z. Poly (N-vinyl-2-pyrrolidone-acrylic acid): comparing of "traditional heating" and "microwave-assisted" free radical polymerization. *J. Chem. Soc. Pak.* **35**, 1191-1196 (2013).
3. Kovac, M., Fuchs, M., Guignard, A., Zufferey, J.C. & Floreano, D. A miniature 7g jumping robot. *IEEE Intl Conf. Robotics and Automation* 373-378 (2008).
4. Bennetclark, H.C. & Alder, G.M. Effect of air resistance on the jumping performance of insects. *J. Exp. Biol.* **82**, 105-121 (1979).
5. Ilton, M. et al. The principles of cascading power limits in small, fast biological and engineered systems. *Science* **360**, eaao1082 (2018).
6. Wensing, P.M. et al. Proprioceptive actuator design in the MIT cheetah: Impact mitigation and high-bandwidth physical interaction for dynamic legged robots. *IEEE Trans. Robot.* **33**, 509-522 (2017).
7. Koh, J.S. et al. Jumping on water: Surface tension-dominated jumping of water striders and robotic insects. *Science* **349**, 517-521 (2015).
